# Ceftazidime-Resistant *Salmonella enterica*, Morocco

**DOI:** 10.3201/eid1510.090247

**Published:** 2009-10

**Authors:** Brahim Bouchrif, Simon Le Hello, Maria Pardos, Bouchra Karraouan, Jean-David Perrier-Gros-Claude, Moulay-Mustapha Ennaji, Mohammed Timinouni, François-Xavier Weill

**Affiliations:** Institut Pasteur du Maroc, Casablanca, Morocco (B. Bouchrif, B. Karraouan, J.-D. Perrier-Gros-Claude, M. Timinouni); Institut Pasteur, Paris, France (S. Le Hello, M. Pardos, F.-X. Weill); Université Hassan II, Mohammedia, Morocco (M.-M. Ennaji)

**Keywords:** Nontyphoidal Salmonella, Salmonella enterica, antimicrobial resistance, extended-spectrum β-lactamases, extended-spectrum cephalosporins, Morocco, food, enteric infections, bacteria, letter

**To the Editor:** Nontyphoidal salmonellosis (NTS) is a major food-borne illness worldwide. Extended-spectrum cephalosporins (ESCs) are currently preferred drugs for treatment of children with NTS. However, resistance to ESCs has emerged worldwide and has become a serious public health problem. This resistance is caused by production of various class A extended-spectrum β-lactamases (ESBLs) and class C cephalosporinases in *Salmonella enterica* (*1*).

National surveillance systems, ideally based on integration of data for animals, food, and humans, are needed to develop strategies for containing antimicrobial drug resistance. Such systems are primarily based on a network of public or private clinical laboratories that refer *Salmonella* isolates to public health laboratories for identification. However, this laboratory-based surveillance system in developing countries is hampered by cost constraints and poor access to quality health facilities, resulting in a low rate of isolation of bacterial pathogens from patients having mild infections. These constraints account for the lack of data and underestimation of the number of NTS cases in many countries, including Morocco.

According to the World Health Organization Global Salm Surv database (www.who.int/salmsurv/activities/en), the Moroccan National Institute of Hygiene reported only 210 human non-Typhi isolates and 999 animal non-Gallinarum isolates during 1999–2003. Antimicrobial drug resistance data are extremely rare. We report the presence of nontyphoidal *Salmonella* isolates resistant to ESCs during an outbreak of food poisoning and in food products in Morocco.

In March 2008, an *S*. *enterica* serotype Typhimurium strain was isolated from stool samples of 45 persons who had attended a wedding ceremony in Errachidia. Clinical symptoms were diarrhea, vomiting, and stomach cramps, beginning 24–72 hours after these persons had eaten a tagine prepared with poorly cooked broiler chickens. Five patients were hospitalized for 3 days, but no deaths were recorded. *S*. *enterica* serotype Typhimurium was isolated from leftovers of a broiler carcass stored in a refrigerator.

PulseNet (http://pulsenetinternational.org/pulsenet/pulsenet.asp) standard pulsed-field gel electrophoresis (PFGE) of *Xba*I-digested chromosomal DNA showed that human and poultry isolates had identical profiles. Antimicrobial drug susceptibility was determined by the disk diffusion method and E-tests, as described (*2*). Isolates were resistant to penicillins and ceftazidime but were susceptible to other antimicrobial drug classes tested. A positive double-disk synergy test result suggested that these strains produced an ESBL. Isolates showed higher levels of resistance to ceftazidime (MIC 128 mg/L) than to ceftriaxone (MIC 8 mg/L).

For identification of the ESBL gene, we conducted PCR amplifications of *bla*_TEM_, *bla*_SHV_, and *bla*_CTX-M_ group genes, as described (*3*). Only the SHV amplicon was obtained, and DNA sequencing showed this amplicon to be 100% identical to *bla*_SHV-12_. Resistance to ESCs and the *bla*_SHV-12_ gene were transferred into *Escherichia coli* by conjugation. An ≈60-kb plasmid was isolated from *E*. *coli* transconjugants and the parental strain. PCR-based replicon typing analysis identified replicon IncI1 (*4*). Although the broilers had been reared locally, no environmental investigation was conducted.

In November 2007, an *S*. *enterica* serotype Newport strain was isolated from a pastry made with locally produced eggs during a food survey conducted in southern Morocco. The isolate was resistant to penicillins, cefoxitin (MIC 128 mg/L), ceftriaxone (MIC 64 mg/L), ceftazidime (MIC 128 mg/L), streptomycin, sulfonamides, chloramphenicol, and tetracycline. We identified the *bla*_CMY-2_ gene carried by a 210-kb nonconjugative plasmid of replicon IncA/C. These CMY-2–producing isolates are also known as *Salmonella* Newport multidrug-resistant (MDR)–AmpC. *Xba*I-PFGE showed a profile similar to the New8a profile described in 2003 in France during a small outbreak linked to consumption of imported horse meat (*2*).

ESC-resistant *Salmonella* isolates have been reported in Morocco (*5*). This report described a serotype Typhimurium clone that produced TEM-3 that was isolated from 10 children hospitalized in Casablanca in 1994. Few studies have been conducted on ESC-resistant *Salmonella enterica* in northern Africa, and most have reported hospital-acquired infections (*1*). Our study identified ESC-resistant *Salmonella* strains in the human food chain and in poultry for human consumption in Morocco. *Salmonella* isolates resistant to ESCs were not identified in food during 2002–2005 at the Institut Pasteur de Casablanca (104 *Salmonella* isolates from 11,516 food samples) (*6*). Emergence in poultry and humans of an MDR serotype Keurmassar strain that produced SHV-12 was reported in Senegal in 2001 (*7*).

Although CMY-2 was originally identified in a serotype Senftenberg isolate from a child in Algeria (*8*), we report the *Salmonella* Newport MDR-AmpC strain in Africa. *Salmonella* Newport MDR-AmpC isolates were reported in 1998 in the United States (*9*), where they quickly spread to cattle and humans. Recent potential spread of this strain into poultry in the United States was suggested by Varma et al. (*10*). Because of the risk for spreading, an efficient national antimicrobial drug resistance monitoring system for foodborne pathogens in Morocco is required to prevent dissemination of bacterial strains resistant to first-line antimicrobial drugs in humans.
